# 740. Elucidation of the Mode of *Clostridioides difficile* Transmission Based on One Health Approach

**DOI:** 10.1093/ofid/ofab466.937

**Published:** 2021-12-04

**Authors:** Sadako Yoshizawa, Tomoka Sawa, Kohji Komori, Masakazu Sasaki, Nobuaki Mori, Kotaro Aoki, Yoshikazu Ishii, Kazuhiro Tateda

**Affiliations:** 1 Toho University School of Medicine; 2 Tokyo Medical Center, Meguro-ku, Tokyo, Japan; 3 Microbiology and Infectious Disease, Ota-ku, Tokyo, Japan; 4 Toho University, Tokyo, Japan

## Abstract

**Background:**

Community-onset *Clostridioides difficile* (*C. difficile*) infection (CACDI) has been increasing in recent years. To explore the transmission route of CACDI, we performed the whole-genome sequencing of *C. difficile* isolated from CACDI patients and compared it to the isolates from livestock, companion animals, and soil.

**Methods:**

From October 2020 until April 2021, fecal specimens of cattle, poultry, swine, felines, canines, CACDI patients, their families, and soil from the CACDI patients' living environment were applied for isolation of *C.difficile*. Whole-genome sequencing of *C. difficile* was performed on the MiSeq system (Illumina). Using the draft genome obtained from these analyses, the house-keeping gene (*tpi*), MLST, toxin genes (*tcdA, tcdB, cdtA, cdtB*), and resistance genes (*gyrA, gyrB, rpoA, rpoB, rpoC*) were comprehensively analyzed.

**Results:**

As of March 31, 2021, 275 specimens were collected. Forty-five fecal specimens of companion animal origin (23 feline and 22 canines) were collected and the positive rate of *C.difficile* was 28.9% (2 felines, 11 canines). In MLST analysis, ST 15 (4 strains), ST 26 (2 strains), ST 42, ST 3, ST 28, ST 100, and ST 185 were detected in canines, and ST 203 and ST 297 strains were detected in felines. Samples of livestock origin were collected from 135 cattle, 41 poultries, and 20 swine. The detection rate in cattle was 11%, toxin-gene positivity was 60%. MLST analysis of 9 strains revealed ST 11 (5 strains), ST 2, ST 15, ST 58, and ST 101. No isolates were found from poultry or swine. Patient-derived strains of CACDI were collected from 14 patients at 2 sites. MLST analysis revealed ST42, ST37, ST100, and ST203(two isolates, respectively), ST 224, ST 81, ST 28, and ST 47. 2 isolates were unclassifiable. One case was a healthy 1-year-old girl, whose family revealed no isolation of *C.difficile*. Impressively, the soil in the parks (A and B) related to the child detected *C.difficile* from 4/4 samples (toxin-gene positivity; 75%) in Park A and 1/4 samples (toxin-gene positive) in Park B. MLST analysis demonstrated ST 42, the same as that in the affected child and core-genome single-nucleotide polymorphisms(SNPs) analysis suggested closely related strain.

**Conclusion:**

Our results suggest one health approach is fundamental to prevent the transmission of *C.difficile*.

**Disclosures:**

**All Authors**: No reported disclosures

